# MicroRNA-mRNA regulatory networking fine-tunes the porcine muscle fiber type, muscular mitochondrial respiratory and metabolic enzyme activities

**DOI:** 10.1186/s12864-016-2850-8

**Published:** 2016-08-02

**Authors:** Xuan Liu, Nares Trakooljul, Frieder Hadlich, Eduard Muráni, Klaus Wimmers, Siriluck Ponsuksili

**Affiliations:** Leibniz Institute for Farm Animal Biology (FBN), Institute for Genome Biology, Wilhelm-Stahl-Allee 2, 18196 Dummerstorf, Germany

**Keywords:** Muscle, Mitochondrial respiratory activity, miRNA-mRNA network

## Abstract

**Background:**

MicroRNAs (miRNAs) are small non-coding RNAs that play critical roles in diverse biological processes via regulation of gene expression including in skeletal muscles. In the current study, miRNA expression profile was investigated in longissimus muscle biopsies of malignant hyperthermia syndrome-negative Duroc and Pietrain pigs with distinct muscle metabolic properties in order to explore the regulatory role of miRNAs related to mitochondrial respiratory activity and metabolic enzyme activity in skeletal muscle.

**Results:**

A comparative analysis of the miRNA expression profile between Duroc and Pietrain pigs was performed, followed by integration with mRNA profiles based on their pairwise correlation and computational target prediction. The identified target genes were enriched in protein ubiquitination pathway, stem cell pluripotency and geranylgeranyl diphosphate biosynthesis, as well as skeletal and muscular system development. Next, we analyzed the correlation between individual miRNAs and phenotypical traits including muscle fiber type, mitochondrial respiratory activity, metabolic enzyme activity and adenosine phosphate concentrations, and constructed the regulatory miRNA-mRNA networks associated with energy metabolism. It is noteworthy that miR-25 targeting *BMPR2* and *IRS1*, miR-363 targeting *USP24*, miR-28 targeting *HECW2* and miR-210 targeting *ATP5I*, *ME3*, *MTCH1* and *CPT2* were highly associated with slow-twitch oxidative fibers, fast-twitch oxidative fibers, ADP and ATP concentration suggesting an essential role of the miRNA-mRNA regulatory networking in modulating the mitochondrial energy expenditure in the porcine muscle. In the identified miRNA-mRNA network, a tight relationship between mitochondrial and ubiquitin proteasome system at the level of gene expression was observed. It revealed a link between these two systems contributing to energy metabolism of skeletal muscle under physiological conditions.

**Conclusions:**

We assembled miRNA-mRNA regulatory networks based on divergent muscle properties between different pig breeds and further with the correlation analysis of expressed genes and phenotypic measurements. These complex networks relate to muscle fiber type, metabolic enzyme activity and ATP production and may contribute to divergent muscle phenotypes by fine-tuning the expression of genes. Altogether, the results provide an insight into a regulatory role of miRNAs in muscular energy metabolisms and may have an implication on meat quality and production.

**Electronic supplementary material:**

The online version of this article (doi:10.1186/s12864-016-2850-8) contains supplementary material, which is available to authorized users.

## Background

MicroRNAs (miRNAs) are endogenous small non-coding RNAs ~22 nt in length that play critical roles in diverse biological processes via epigenetic regulation of gene expression. Precursor miRNAs (pre-miRNA) are initially generated in nucleus and processed into an approximately 70 nt long stem-loop structure. It is then exported to cytoplasm and processed by Dicer to generate miRNA/miRNA duplexes. One strand of which is incorporated with Agonaute to form RNA-Induced Silencing Complex (RISC) that targets mRNAs via base-pair complementary, typically to their 3′ untranslated regions (3′UTR) or CDs and downregulates gene expression by either degradation of mRNA or repression of translation, while the other strand is usually discarded [1].

Skeletal muscle is highly metabolically active and valuable for meat-producing animals. Slow-twitch-oxidative (STO), fast-twitch-oxidative (FTO), and fast-twitch-glycolytic (FTG) fiber were the three major muscle fiber types in pigs. Muscle fibers have strong association with muscle metabolic activities and meat quality such as tenderness, juiciness and color. Muscle containing a high proportion of oxidative fibers is often associated with higher fat content, oxidative enzyme activities and mitochondrial density [2–4]; a high ratio of FTG fibers is associated with high glycolytic enzyme activities. Previous research has identified several miRNAs are associated with meat quality such as miR-133, miR-221 and miR-103 etc. in porcine skeletal muscle [5]. The polymorphisms in the porcine miR-133 and miR-208 are proposed as a genetic factor affecting muscle fibers and meat quality traits [6, 7]. Since the critical roles of miRNAs such as myogenesis, adipogenesis and muscle development have been discovered in pig skeletal muscle [8–11], the understanding of the miRNA regulation in metabolic properties of skeletal muscle fibers could be key to improvement of meat quality [12]. MiR-210 and miR-338 could regulate the gene expression of oxidative phosphorylation (OXPHOS) machinery including complex IV subunits COX10, COXIV and ATP synthase subunits ATP5G1 [13, 14]. MiR-15a and miR-15b modulate the cellular ATP levels [15, 16]. MiR-696 regulates the fatty acid oxidation and mitochondrial biogenesis through targeting peroxisome proliferator-activated receptor-gamma coactivator-1alpha (PGC-1α) [17]. With those miRNAs identified, the lack of a comprehensive and systematic miRNA profiling associated with energy metabolism of skeletal muscle remains unraveled.

Our previous research on muscle transcriptional profile has revealed numerous biological pathways significantly associated with muscle fiber type, mitochondrial respiratory activity and metabolic enzymes [18]. It is of interesting to further investigate how miRNAs are involved in energy metabolism by fine-turning gene expression. In the present study, the miRNA transcriptome profiling of longissimus muscle (LM) samples obtained 24 h before slaughter of two pig breeds Duroc and Pietrain exhibiting divergent meat quality and muscle phenotypes provided a comprehensive insight into the discovery of miRNAs associated with muscle fiber, mitochondrial respiratory activity and metabolic enzyme activity. Muscle of Duroc pigs contains higher percentage of STO fibers, mitochondrial respiratory activity and higher fat content to improve the tenderness and juiciness of the meat. In comparison, PiNN pigs are more muscular and favorable for meat industry. Their skeletal muscles are leaner and contain more FTG fibers [18–20]. Hence Duroc and Pietrain pigs are great models to study energy metabolism of skeletal muscle. The miRNA and mRNA expression profile was then integrated based on their pairwise correlations and computational target prediction to construct the regulatory miRNA-mRNA networks which could potentially affect metabolic properties of skeletal muscle and hence meat quality. The illumination of miRNA-based regulatory metabolism could enrich our knowledge of the roles of miRNAs in achieving phenotypic diversity of skeletal muscle in different breeds.

## Methods

### Animals and sample collection

The experiment and muscle biopsy collection were approved and authorized by the German and European animal welfare regulations for animal husbandry, transport, and slaughter [19–21]. All experimental procedures, including animal care and tissue sample collection, followed guidelines for safeguarding and good scientific practice in accordance with the German Law of Animal Protection, officially authorized by the Animal Care Committee and authorities [Niedersächsischen Landesamt für Verbraucherschutz und Lebensmittelsicherheit (LAVES) 33.42502/01-47.05].

As previously described [19–21], Duroc and Pietrain (PiNN) pigs were raised until 180 days of age. To rule out the effects of the maglinant hyperthermia syndrome (MHS) locus, only muscle samples from MHS-negative genotype pigs were investigated. Muscle biopsies were collected from five female and male pigs of each breed (*n* = 20) for phenotypic measurements (see Additional file 1 for detailed phenotype definition and measurement) [18–21]. Biopsies were collected 24 h before slaughter from the longissimus muscle between 13th and 14th thoracic vertebrae. Muscle samples were frozen in liquid nitrogen and stored at −80 °C until analysis.

### RNA isolation

Small RNAs were isolated and enriched from longissimus muscle biopsies using a miReasy Mini kit and an RNeasy MinElute Cleanup kit (Qiagen) according to manufacturer’s protocols. The quality and quantity of small RNA were assessed with an Agilent 2100 Bioanalyzer (Agilent) using an Agilent small RNA kit.

### MicroRNA microarray analysis

The Affymetrix GeneChip miRNA 3.0 Array (Affymetrix) was used to determine the miRNA expression profile of the LM 24 h ante mortem of Duroc and PiNN pigs. It is comprised of 16,772 entries representing hairpin precursor, total probe set 19,724 for detection of most miRNA from 153 species (miRBase V.17). 200 ng of small RNA were used in sample preparation using a FlashTag Biotin HSR RNA Labeling kit (Genisphere) for Affymetrix GeneChip miRNA Arrays. Each of the labeled RNA (*n* = 20) was then hybridized for 16 h to an Affymetrix GeneChip miRNA array according to manufacturer’s recommendation (Affymetrix), washed and stained in the Affymetrix Fluidics Station 450, and scanned on the Affymetrix G3000 Gene Array Scanner. Expression Console software was used for robust multichip average (RMA) normalization and the detection of present miRNAs by applying the DABG (detection above background) algorithm. Further filtering was done by excluding probes that were present in less than 70 % of the samples within each breed and annotated miRNAs that had sequence greater than or equal to 30 nt in length. Three thousand five hundred eighty seven probes passed the quality filtering and were used for further analysis. The availability of expression data are in the Gene Expression Omnibus public repository with the GEO accession number GSE80198: GSM2120718-GSM2120737.

### Statistics and bioinformatics analyses

Differential expression analysis for miRNA was performed using the ANOVA procedure in JMP genomics 7(SAS Institute). Breed was treated as a fixed effect. False discovery rate (FDR) was used to control an error rate of a multiple-hypothesis testing according to Benjamin & Hochberg [22]. We used our previous microarray-based mRNA expression data to integrate with the differentially expressed miRNAs and scan for potential target genes. Pearson correlation of miRNA and mRNA expression levels was calculated.

Both RNAhybrid 2.1.2 and TargetScan 7.0 were used to predict targets of miRNAs. RNAhybrid (http://bibiserv.techfak.uni-bielefeld.de/rnahybrid) is computational software that detects the most energetically favorable hybridization sites of a small RNA within a large RNA [23, 24]. The miRNA probe sets were tested with the following parameters: number of hits per target = 1 and energy cutoff = −25 kcal/mol and maximal internal or bulge loop size per side = 4. TargetScan (http://targetscan.org/) was used to predict the target gene candidates based on complementarity of the miRNA seed sequence (position 2-8 of the miRNA 5′-end) and target binding site on the 5′ UTR, 3′ UTR and protein coding region of the porcine mRNA sequences (Sus scrofa 10.2 download from NCBI: http://www.ncbi.nlm.nih.gov/ on 1.9.2015) [25]. Hafner et al. and Chi et al. have found that Argonaute-bound target sites in coding sequences (CDs) are as numerous as those located in 3′ UTR in both HEK293 cells (50 % CDs vs 46 % 3′UTR) [26] and mouse brain (25 % CDs vs 32 % 3′UTR) [27]. Other research suggests that miRNA target sites in 3′UTR are more efficient at triggering mRNAs degradation while CDs and 5′UTR located sites can effectively repress translation [28, 29]. Xu et al. develop novel computational approach for target prediction with sites located along the entire gene sequences to increase the percentage of true positive targets [30]. Hence 5′ UTR, CDs and 3′UTR were included in this study to improve the sensitivity of miRNA target identification and avoid a substantial number of missing targets. Transcripts that negatively correlated with miRNA and predicted as potential targets were further passed to functional analysis.

IPA software (Ingenuity System, https://www.ingenuity.com) was used for functional analysis of potential miRNA target genes. It categorizes genes based on annotated gene functions and statistically tests for over-representation of functional terms within the gene list using Fisher’s Exact Test. The miRNA-mRNA regulatory networks were visualized using Cytoscape V3.2.1 (http://cytoscape.org) [31].

### Quantitative real time PCR (qPCR) for microRNA microarray (miChip) validation

Four miRNAs (ssc-miR-24-3p, ssc-miR-30a-5p, ssc-miR-126 and ssc-miR-145) related to energy metabolism were validated by qPCR of each individual sample (*n* = 20). The same samples were used for both qPCR validation and miRNA-chips. The cDNA was synthesized from 250 ng isolated miRNAs using an NCode^TM^ VILO^TM^ miRNA cDNA Synthesis Kit (Invitrogen) according to manufacturer’s protocols. Real-time PCR was performed using the EXPRESS SYBR GreenER^TM^ miRNA qRT-PCR Kit with premixed ROX (Invitrogen) according to manufacturer’s protocols. All measurements were performed in duplicates. The thermal parameters were 50 °C for 2 min, 95 °C for 2 min, followed by 45 cycles of 95 °C for 15 s and 60 °C for 1 min. The universal qPCR primer was provided in the kit and the miRNA-specific forward primers were designed for the miRNAs of interest. The designed primer sequence information is accessible in Additional file 2. Geometric mean of the 5S and U6 transcription levels was used as an internal standard to normalize the miRNA expression value. Correlation coefficient analysis between the miChip and qPCR data was calculated using SAS 9.3 (SAS Institute).

## Results

### Differentially expressed miRNAs between Duroc and Pietrain

Out of 3587 probes quality-filtered probes, 58 probes belonged to 27 unique mature miRNA sequences were differentially expressed (*p* < 0.05, FDR < 0.2) between Duroc and PiNN using ANOVA on JMP Genomics 7. Among these, 33 probes belonged to 8 mature miRNA sequences were up-regulated in Duroc pigs, while 25 probes belonged to 19 different mature miRNA sequences were up-regulated in PiNN pigs (Additional file 3). Among these, miR-363, miR-423 and miR-34 were the top three upregulated miRNAs in Duroc pigs with fold change ranging from 3.29 to 1.68. Whereas the top three upregulated miRNAs in PiNN were miR-4687, miR-3619 and miR-22 with fold change ranging from 3.45 to 2.71.

### Correlation between differentially expressed miRNAs and target mRNAs

**Fig. 1:**
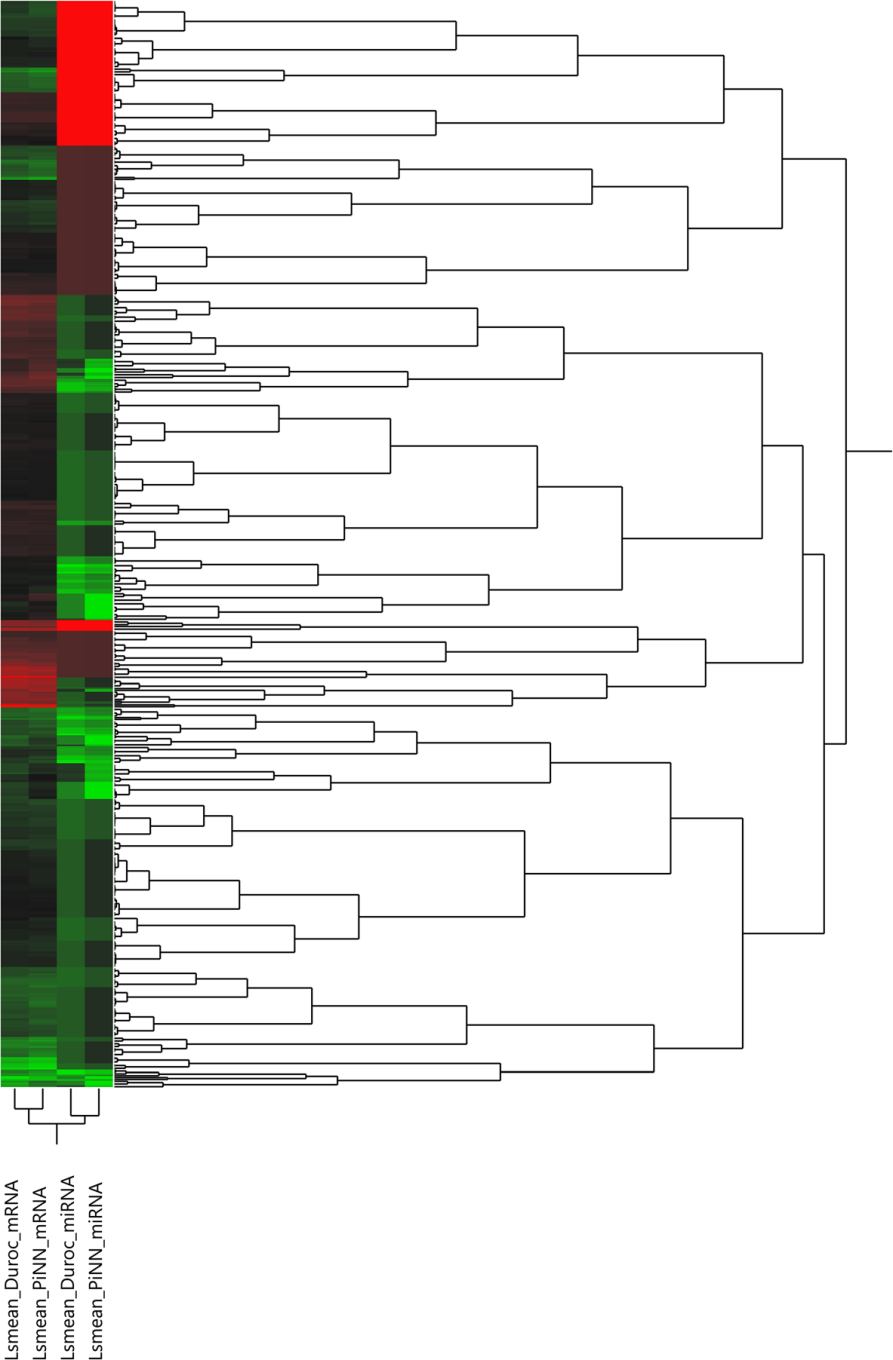
Heatmaps and hierarchical cluster of mRNA and miRNA pairs based on Least squares (Ls) means of differentially expressed mRNA and miRNA (FDR < 0.05) between breed Duroc and PiNN

**Fig. 2:**
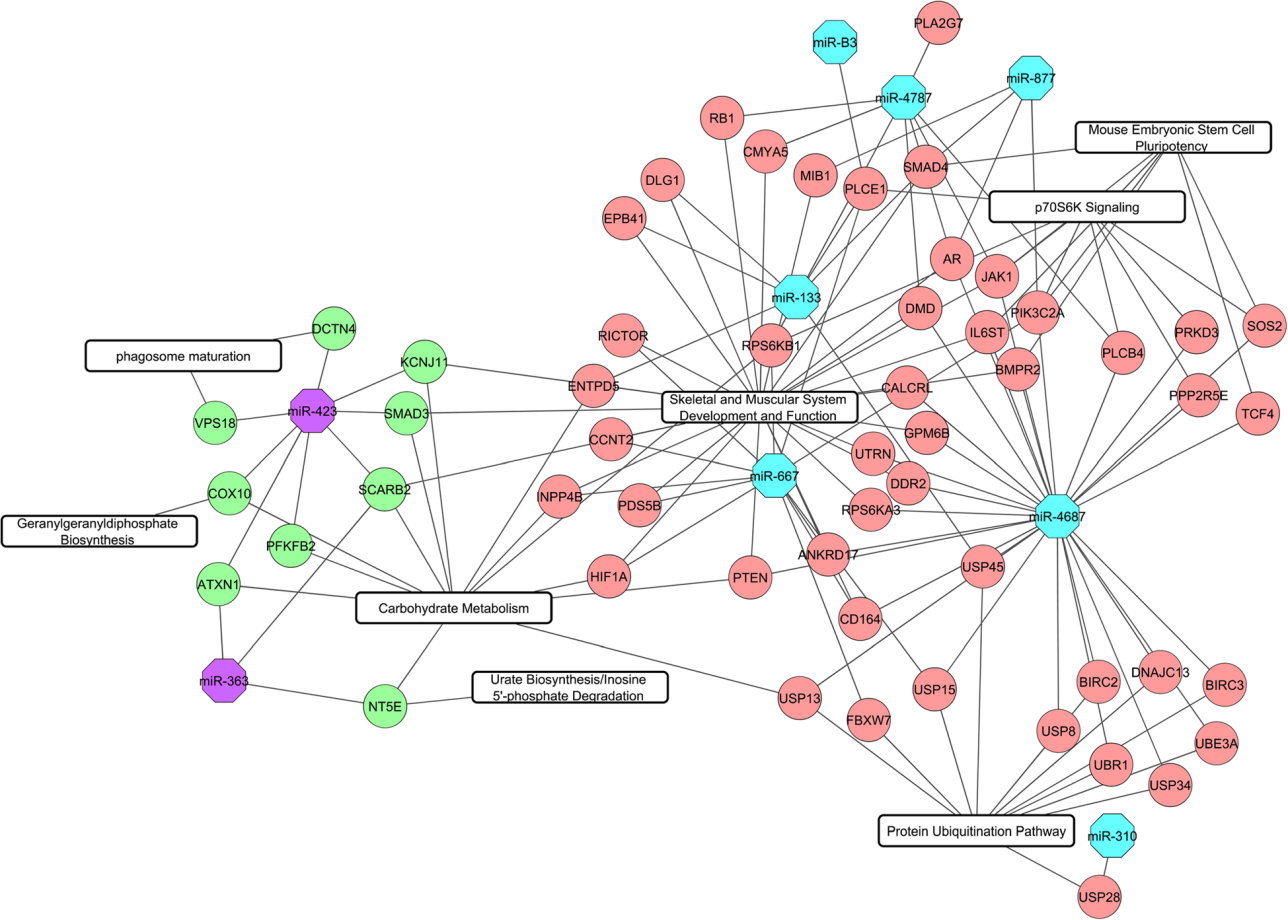
Differentially expressed miRNA-mRNA pairs and regulatory network between breed Duroc and PiNN. *Blue* indicates miRNAs with higher expression in PiNN, *purple* indicates miRNAs with higher expression in Duroc, *red* indicates genes with higher expression in Duroc and *green* indicates genes with higher expression in PiNN

The mRNA expression data of matched samples from our previous study (GEO accession number GSE69840: GSM1709900 - GSM1709919) were used for a pairwise correlation analysis [18]. In total, 2345 mRNA probes were differentially expressed (*p* < 0.05, FDR < 0.05) between two breeds. A pairwise correlation coefficient analysis was computed between 58 miRNA probes and 2345 mRNA probes. Among the 136,010 Pearson correlation coefficients, 12,408 negative correlated miRNA-mRNA pairs were detected at *p* < 0.05 and FDR < 0.05 for correlation between miRNA and mRNA. Computational target prediction was performed using Targetscan and RNA hybrid. After combining the correlation analysis and target prediction results, 598 miRNA-mRNA pairs containing 340 genes and 11 mature miRNA sequences were retained (Additional file 4). The heat map and hierarchical clustering of miRNA-mRNA pairs based on their expression levels (lsmeans) shown in Fig. 1 demonstrates an inverse relationship between mRNAs and mRNA target candidates. All the target genes were further analyzed with IPA to identify prominent functions and pathways that may contribute to divergent muscle metabolic properties between the two breed types. Target genes assigned to the functional categories related to skeletal and muscular system development and function as well as carbohydrate metabolism were focused on. The top three canonical pathways for Duroc up-regulated target genes were protein ubiquitination pathway, p70S6K signaling and mouse embryonic stem cell pluripotency, while geranylgeranyl diphosphate biosynthesis, phagosome maturation and urate biosynthesis/Inosine 5′-phosphate degradation for PiNN up-regulated target genes. A representative miRNA-mRNA regulatory network of focused biological pathways depicted in Fig. 2 illustrates a complex relationship and networking of the two biomolecule types.

### Correlation between miRNA expression and phenotypic traits

The expression of 3587 miRNA probes was calculated for the correlation with traits of muscle fiber composition, mitochondrial respiratory activity and metabolic enzyme activity in both Duroc and PiNN pigs. In total, 3263 miRNA-phenotype pairs containing 1864 miRNA probes belonged to 757 mature miRNA sequences were identified at *p* < 0.05 shown correlation between miRNA and at least one of the 19 phenotypes. The top 100 significant miRNAs correlated to phenotypes were shown in Additional file 5 (*p* < ~0.001). Table 1 showed the top five miRNAs significantly correlated to each phenotype.

**Table 1:** The top five significant miRNAs correlated to phenotypes in both Duroc and PiNN pigs

Phenotype	Top five significant miRNAs	*P*-value	|Correlation|
STO	miR-130, miR-208, miR-363, miR-24, miR-126	3.123E-04–1.195E-03	0.803–0.753
FTO	miR-22, miR-166, miR-1892, miR-1343, miR-143	1.222E-03–4.514E-03	0.752–0.689
FTG	miR-99, miR-499, let-7, miR-181, miR-154	1.577E-04–3.337E-04	0.824–0.801
State3_pyruvate	miR-30, miR-196, miR-190, miR-363, miR-95	1.508E-03–7.169E-03	0.661–0.581
State3_succinate	miR-126, miR-196, miR-363, miR-1892, miR-30	7.329E-04–4.205E-03	0.691–0.611
State4_CAT	miR-196, miR-2012, miR-192, miR-202, miR-17	2.484E-04–1.360E-02	0.731–0.542
RCI_pyruvate	miR-17, miR-182, miR-203, miR-27, miR-378	6.558E-03–1.258E-02	0.587–0.547
RCI_succinate	miR-182, miR-203, miR-17, miR-27, miR-542	3.630E-04–4.034E-03	0.718–0.613
GP	miR-148, let-7, miR-194, miR-7, miR-25	3.063E-03–1.477E-02	0.627–0.536
PFK	miR-130, miR-208, miR-193, miR-32, miR-24	5.944E-04–1.455E-03	0.700–0.663
LDH	miR-17, miR-58, miR-15, miR-769, miR-345	1.090E-04–4.384E-03	0.758–0.609
CS	miR-338, miR-30, miR-17, miR-182, miR-455	3.970E-05–1.959E-03	0.786–0.649
ComplexI	miR-182, miR-181, miR-143, miR-28, miR-765	2.691E-04–1.620E-03	0.743–0.672
ComplexII	miR-182, miR-1, miR-1307, miR-203, miR-499	2.557E-03–2.458E-02	0.666–0.527
ComplexIV	miR-196, let-7, miR-451, miR-128, miR-99	3.668E-03–7.785E-03	0.618–0.577
IMP	miR-168, miR-166, miR-58, miR-223, let-7	3.908E-04–1.382E-02	0.745–0.569
AMP	miR-10, miR-126, let-7, miR-27, miR-450	9.860E-05–5.804E-04	0.789–0.730
ADP	miR-15, miR-885, miR-322, miR-450, miR-338	1.316E-04–4.540E-03	0.781–0.636
ATP	miR-15, miR-450, miR-210, miR-885, miR-451	4.811E-04–9.562E-03	0.737–0.593

### Integration of correlated miRNAs, mRNAs and phenotypic traits

**Fig. 3:**
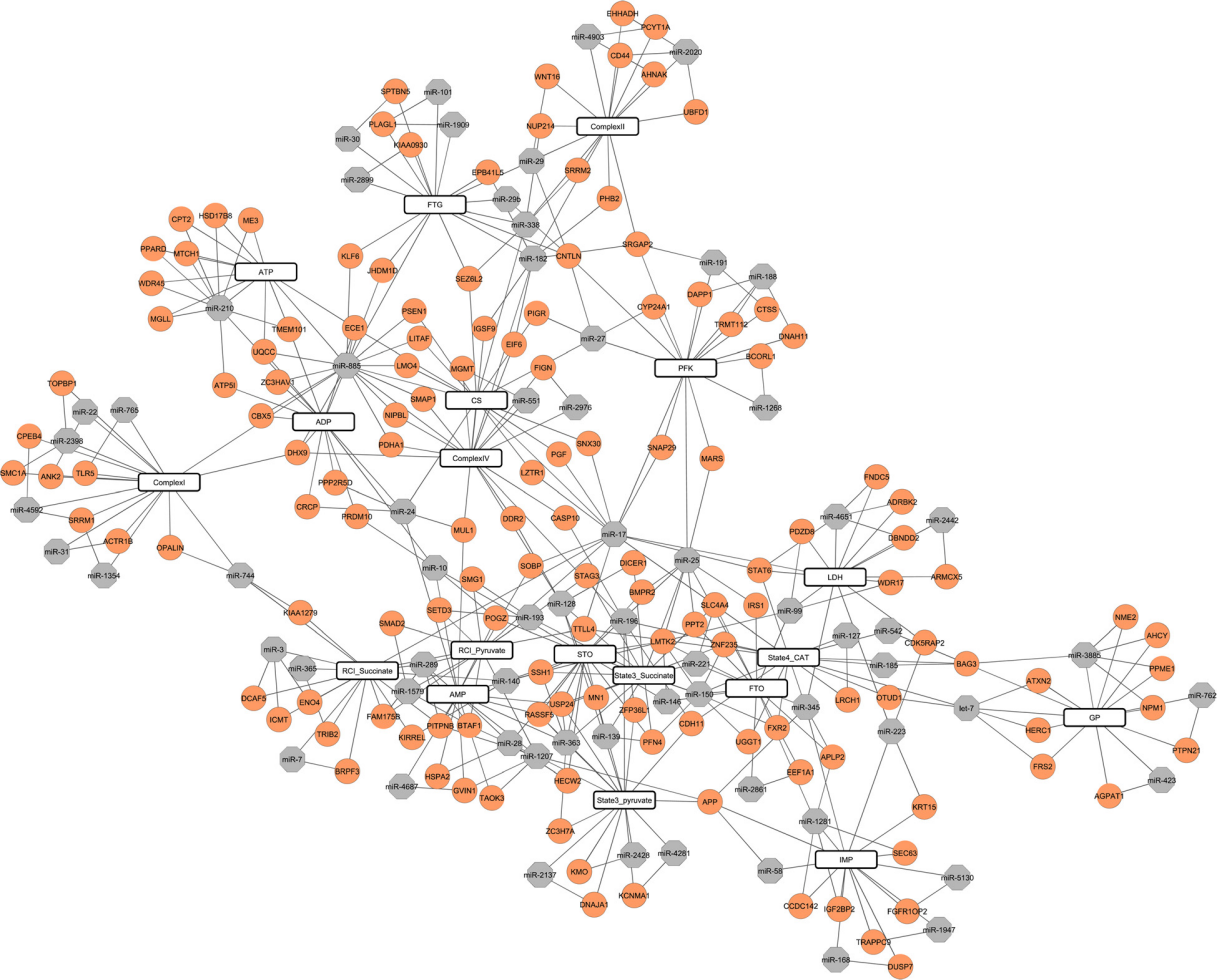
Regulatory network of miRNA-mRNA associated with muscle fiber composition, mitochondrial respiratory activity, metabolic enzyme activity and adenine nucleotide concentration for breed Duroc and PiNN. Genes were colored with *orange* while miRNAs with *grey*

Correlations between gene expression derived from post quality-filtered 17,820 mRNA probes and each phenotypic- trait were calculated for both Duroc and PiNN pigs. In total, 24,374 mRNA-phenotype pairs containing 11,091 mRNA probes belonging to 7489 genes were identified at *p* < 0.05. The top 100 significant mRNAs correlated with phenotypes are accessible in Additional file 6 (*p* < 0.0002). Pairwise correlation coefficient analysis was then performed between the identified 1864 miRNA probes and 11,091 mRNA probes which correlated with at least one of the 19 phenotypes. After combining with the target prediction results, 26,861 miRNA-mRNA pairs containing 3182 genes and 387 miRNAs (*p* < 0.05) were identified to correlate with at least one phenotype. The top ten miRNA-mRNA pairs for each phenotype were shown in Fig. 3 and Additional file 7 (*p* < 0.05, FDR < 0.24).

### Correlation relationship between mitochondrial and UPS related genes

From all identified top 10 miRNA-mRNA pairs associated with each phenotypic trait (Fig. 3), the expression correlation between 9 selected nuclear-encoded mitochondrial-related genes and 7 selected UPS-related genes were calculated. In Table 2, mitochondria related genes: ATP synthase, mitochondrial F0 complex, subunit E (*ATP5I*), Malic enzymes 3 (*ME3*), mitochondrial carrier 1 (*MTCH1*), cytochrome P450, family 24, subfamily A, peptide 1 (*CYP24A1*), kinesin family member 1 binding protein (*KIAA1279*), prohibitin 2 (*PHB2*), pyruvate dehydrogenase alpha 1 (*PDHA1*) and ubiquinol-cytochrome c reductase complex chaperone (*UQCC*) were significantly correlated with at least one of the six UPS-related genes: Sus scrofa similar to E3 ubiquitin-protein ligase HECW2 (*HECW2*), ubiquitin specific peptidase 24 (*USP24*), ubiquitin family domain containing 1 (*UBFD1*), mitochondrial ubiquitin ligase activator of NFKB 1-like (*MUL1*), amyloid beta (A4) precursor protein (*APP*) and heat shock 70 kDa protein 2 (*HSPA2*) at *p* < 0.05.

**Table 2:** Correlation between mitochondrial and UPS related gene expressions

Genes			Mitochondria related genes							
			*ATP5I*	*ME3*	*MTCH1*	*CYP24A1*	*KIAA1279*	*PHB2*	*PDHA1*	*UQCC*
UPS related genes	*HECW2*	*r*	0.386	0.623	0.494	−0.318	−0.392	−0.306	0.586	0.509
		*p*	0.093	**0.003**	**0.027**	0.171	0.087	0.189	**0.007**	**0.022**
	*USP24*	*r*	−0.285	−0.284	−0.376	0.160	0.542	0.478	−0.422	−0.281
		*p*	0.224	0.226	0.102	0.501	**0.014**	**0.033**	0.064	0.231
	*UBFD1*	*r*	0.512	0.501	0.746	0.069	−0.512	−0.669	0.477	0.515
		*p*	**0.021**	**0.024**	**0.0002**	0.772	**0.021**	**0.001**	**0.034**	**0.020**
	*MUL1*	*r*	0.336	0.083	0.131	0.657	0.460	0.224	−0.375	0.051
		*p*	0.147	0.726	0.583	**0.002**	**0.041**	0.341	0.103	0.832
	*APP*	*r*	0.214	0.486	0.463	−0.368	−0.300	−0.292	0.214	0.327
		*p*	0.365	**0.030**	**0.040**	0.110	0.199	0.211	0.364	0.159
	*HSPA2*	*r*	0.414	0.484	0.566	−0.212	−0.502	−0.474	0.537	0.274
		*p*	0.070	**0.031**	**0.009**	0.369	**0.024**	**0.035**	**0.015**	0.242

numbers in bold are *p*-values < significance threshold of 0.05

### qRT-PCR validation

**Fig. 4:**
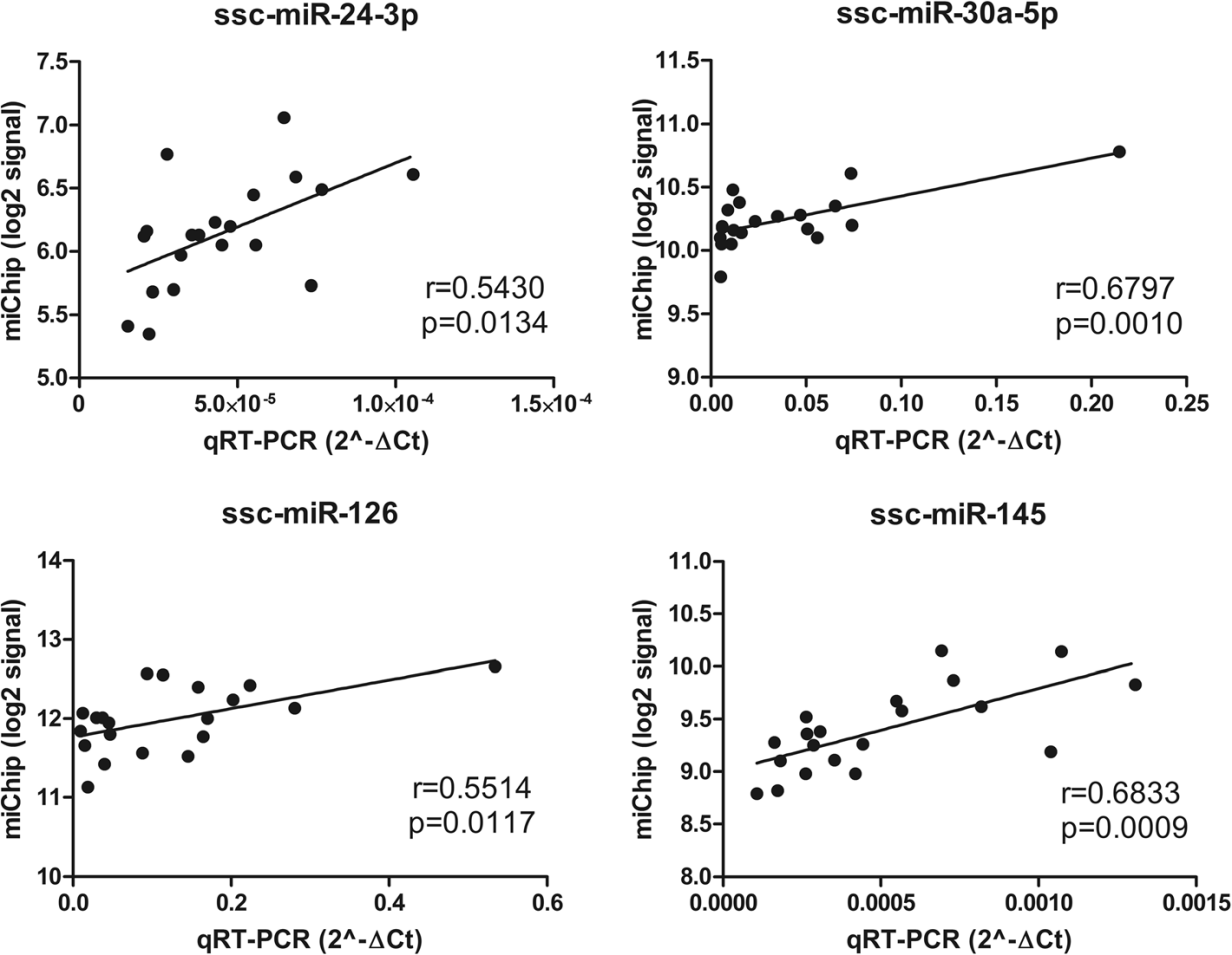
qPCR validation of miChip results for four microRNAs: ssc-miR-24-3p, ssc-miR-30a-5p, ssc-miR-126 and ssc-miR-145. Plot between qPCR (2^-∆Ct on the x-axis) and miChip (log2 signals on the y-axis) for each miRNA. The corresponding correlation coefficient (r) and *p* values are shown

The expression of ssc-miR-24-3p, ssc-miR-30a-5p, ssc-miR-126 and ssc-miR-145 were random selected for validation by qRT-PCR. The correlation coefficient between qPCR and miChip data ranged from 0.543 (*p* = 0.0134) to 0.6833 (*p* = 0.0009), suggesting a good concordance between miChip and qPCR results, as shown in Fig. 4.

## Discussion

Duroc and Pietrain are divergent for different muscle characteristics and meat quality. Duroc pigs are fattier and their skeletal muscle contains larger amount STO fibers which are generally associated with higher oxidative enzyme activities, mitochondrial respiratory activity and the storage of lipid to improve the tenderness and juiciness of the meat, whereas Pietrain pigs are more muscular and their muscles are more lean and contain higher percentage of FTG fibers which are associated with higher glycolytic enzyme activities [2, 18–20, 32, 33]. The higher percentage of FTG fibers may result in lower capillarization, insufficient delivery of oxygen and glycogen depletion, which ultimately lead to dry, firm, and dark meat [34, 35]. Since microRNAs have been known as critical regulators in energy metabolism of skeletal muscle, in the present study, miRNA expression profiling and functional analysis may shed light on miRNA-based epigenetic regulatory mechanism of muscle fiber type and metabolic enzyme activity and hence may be translated to improvement of meat quality.

The miRNA profile of porcine skeletal muscle has been investigated in several studies and the miRNA-mRNA networks are constructed [36–38]. Hou et al. analyzes the miRNA and mRNA profile for Landrace (lean-type) and Tongcheng (obese-type) pigs [36]. In both the study of Hou et al. and our present study, biological process of muscle development was shown in the identified differentially expressed miRNA-mRNA network and it highlights the importance of the regulatory role of miRNAs in diverging porcine muscle development between obese-type and lean-type pigs. Further, some miRNAs such as miR-363, miR-133 and miR-423 were identified in the network of both studies. Tang et al. investigated the miRNA and mRNA expressions of skeletal muscle for Landrace and Tongcheng pigs at 33, 65 and 90 days to explore prenatal muscle development while Jing et al. construct differentially expressed miRNA-mRNA network between different residual feed intake in pigs [37, 38].

### Roles of the differentially expressed miRNAs and their target genes in divergent muscle characteristics

To understand the regulatory role of miRNAs that may contribute to phenotypic variation of the skeletal muscle in different pig breeds, differentially expressed miRNAs and mRNAs between the two breeds were integrated, so that the key target genes regulated by key miRNAs were identified. Functional analysis results showed that the target genes were significantly enriched for various muscle related bio-functions suggesting biological related results rather than random noise. The top canonical pathway for Duroc-up regulated genes was protein ubiquitination pathway containing genes of *USP28*, *USP8*, *USP45*, *USP15*, *USP13*, *UBR1*, *DNAJC13*, *FBXW7*, *BIRC3*, *USP34*, *UBE3A*, BIRC2. The ATP-dependent ubiquitin is mediated by ubiquitin-activating enzyme E1, specific ubiquitin-conjugating-enzyme E2 and ubiquitin protein E3 to promote protein degradation via the 26 s proteasome and has implications on meat quality and muscle atrophy [39–42]. In our study, *USP45* and *USP28* were predicted as a direct target of miR-133 and miR-310 respectively. MiR-133b could influence both major apoptosis pathways and wound healing [43, 44], and most importantly, the polymorphisms in the porcine MiR206/MiR133b cluster are proposed as a genetic factor affecting muscle fibers and meat quality traits [6]. MiR-311-3p belongs to miR-310 miRNA family and its loss of function can cause defects in energy metabolism and deregulation of nutritional homeostasis-associated genes [45]. MiR-363 has been discovered as a negative regulator of adipogenesis in adipose tissue-derived stromal cells by directly targeting the 3′UTR of E2F3 [46]. This is in line with our study that the expression level of miR-363 is higher in Duroc than PiNN with average fold change more than 3. Other Duroc up-regulated genes such as *CMYA5*, *AR*, *RB1* and *BMPR2* in functional category of skeletal and muscular system development and function were regulated by miR-4787, miR-877 and miR-4687 etc. Cardiomyopathy associated 5 (*CMYA5*) also called TRIM76 belongs to the tripartite motif super family of proteins (TRIM). Its interaction with both M-band Titin and Calpain 3 suggests its relevance to Limb-girdle Muscular Dystrophies [47]. One SNP (A7189C) of *CMYA5* is significantly associated with water loss and intramuscular fat, which proposes the porcine *CMYA5* as a potential candidate gene for meat quality [48]. Androgen Receptor (AR) is a steroid-hormone activated transcriptional factor. The androgen-AR signaling pathway promotes the slow-twitch muscle fiber formation in skeletal muscle by increasing the expression of slow-twitch-specific genes and suppressing the fast-twitch-specific genes [49].. Moreover, retinoblastoma 1 (RB1) has been identified to be related to Marbling trait in cattle via gene co-expression analysis [50]. Bone Morphogenetic Protein Receptor type II (*BMPR2*) encodes a member of the bone morphogenetic protein receptor family of transmembrane serine/threonine kinases. It is essential for BMP signaling and may be involved in the regulation of adipogenesis and hence in Obesity [51]. All the above Duroc-up regulated genes with their corresponding down regulated miRNAs could contribute to the higher amount of oxidative muscle fibers and fat content. On the other hand, PiNN-up regulated genes *SMAD3* and *PFKFB2* were regulated by miR-423. These two genes are involved in not only muscle fiber specificity but also the promotion of glycolysis in skeletal muscle. SMAD family member 3 (SMAD3) promotes muscle atrophy in vivo by regulating atrogin-1, mTOR and protein synthesis [52]. It suppresses the expression of peroxisome proliferator-activated receptor γ coactivator 1-α (*PGC*-*1α*) [53], which is a master coordinator to control mitochondrial biogenesis and drives the formation of slow-twitch muscle fibers [54, 55]. Fructose -2, 6-biphosphatase 2 (*PFKFB2*) can promote glycolysis by controlling the level of Fructose 2, 6- bisphosphate, which is an allosteric activator of phosphofructokinase (PFK-1) [56]. Collectively, we demonstrate that differential miRNAs and target gene candidates assemble regulatory networks that may fine-tune the expression of genes within the pathways and shape the related phenotypes among pig breeds.

### Phenotype-correlated microRNAs link to various processes in energy metabolism

Numerous studies have revealed the critical roles of miRNAs in skeletal muscle development and the irregular miRNA expression contributes to various muscular disorders [57]. Table 1 shows the top five miRNAs significantly correlated to each phenotypic trait including muscle fiber type, mitochondrial respiratory activity (MRA) and metabolic enzyme activities for Duroc and PiNN pigs. Both positive and negative correlations were included to discover any potential links between miRNAs and phenotypes. STO, FTO, and FTG fibers were three major muscle fiber types in pigs which are strong associated with muscle metabolic activities and meat quality. MiRNAs such as miR-208, miR-499, miR-130 and miR-363 showed highly significant correlation with muscle fiber types. MiR-208 and miR-499 play a dominant role in the specification of muscle fiber identity by inducing the type I fiber program via targeting Sox6 which acts as a repressor of slow-twitch genes [58–60]. MiR-130b belongs to miR-130 family directly targets gene PGC-1α, which is a master regulator for mitochondrial biogenesis and its activation promotes the slow, oxidative myogenic program in mice and drives the formation of slow-twitch muscle fibers in cultured muscle cells [54, 61]. In C2C12 cells, miR-130b could modulate cellular ATP levels by targeting electron transport chain subunits Ndufb7 and Cox6a2 [16]. As previously mentioned, miR-363 has been discovered as a negative regulator of adipogenesis in adipose tissue-derived stromal cells by directly targeting the 3′UTR of E2F3 [46]. Since lipids are stored mainly in STO muscle fibers to improve tenderness and juiciness of the meat [33], and muscles containing more STO fibers are associated with higher oxidative enzyme activities and mitochondrial respiratory activity [2], it is expected that miR-363 shows high correlation with STO muscle fiber type and mitochondrial respiratory activity including state 3 pyruvate and state 3 succinate. MiR-30 family members haven been demonstrated to control calcium signaling by directly inhibiting a Ca^2+^ transporter *TRPC6* etc. [62]. Considering the crucial regulatory role of calcium signaling in mitochondrial ATP production, it was not unexpected that miR-30 was associated with mitochondrial respiratory activity including state3 pyruvate and state3 succinate and TCA involved CS enzyme activity. Furthermore, miR-30 directly targets Prdm1 to promote fast muscle formation since Prdm1 regulates muscle fiber differentiation by repressing the transcription factor Sox6 which acts as a repressor of slow-twitch-specific gene expression [60, 63]. All these reinforced the association between STO fiber, mitochondrial respiratory activity and fat content. MiR-196 was highly correlated to mitochondrial state 3 and state 4 respiration rate and Complex IV activity. MiR-196a displays a tissue-specific expression pattern in porcine and plays a role in porcine adipose development via inducing the expression of adipocyte specific markers, lipid accumulation and triglyceride content [64]. MiR-542 was significantly correlated to RCI succinate. MiR-542-3p directly targets bone morphogenetic protein 7 (BMP7), which induces differentiation of adipose derived mesenchymal stem cells into brown adipocytes and increases mitochondrial activity in mature brown adipocytes [65–67]. MiR-1 was correlated to Complex II activity in the present study. MiR-1 family is abundantly expressed in cardiac and skeletal muscle. It post-transcriptionally represses the expression of genes in antioxidant network and thus influences susceptibility to cardiac oxidative stress of miR-1 transgenic mice [68]. Moreover, it was proposed as a candidate gene associated with muscle fiber type composition [69]. MiR-7, miR-194 and miR-25 were identified to correlate with GP activity. Both miR-7 and miR-194 could directly target and suppress the expression of insulin-like growth factor 1 receptor (IGF-1R) whereas miR-25 regulates insulin synthesis at its mRNA level [70–72]. Since insulin and insulin-like growth factor system are crucial for normal glucose homeostasis [73, 74], it is likely that miR-7 and miR-194 could play a role in glucose metabolism via IGF-1R and insulin. MiR-210, miR-15 and miR-338 were highly correlated to the concentration of ADP and ATP in muscle cells. MiR-210 and miR-338 regulate the expression of oxidative phosphorylation (OXPHOS) machinery including complex IV subunits *COX10*, *COXIV* and ATP synthase subunits *ATP5G1* correspondingly [13, 14]. The fully assemble of OXPHOS system could directly contribute to the ATP production. Furthermore, the modulation of cellular ATP levels by miR-15b was supported by other work as well [15]. Overall, our results and previous reports functionally link miRNAs to muscle fiber specificity, mitochondrial respiration, adipogenesis, glucose metabolism and ATP production and further suggest an essential role of miRNAs in energy metabolism.

### Phenotype-correlated miRNA-mRNA regulatory network link to energy metabolism

Based on the identified miRNAs that highly correlated with the phenotypes, we further integrated the miRNA and mRNA expression profiles to identify miRNAs regulated genes that influence energy metabolism. The microRNA-mRNA regulatory network was constructed using the following criteria: 1) The expressions of both miRNAs and target mRNAs were correlated to the phenotypical traits 2) The gene expression level was negatively correlated with the expression of its miRNA regulator 3) The gene was computationally predicted as a target gene of the corresponding miRNA. It is noteworthy that miR-25 together with its target genes Bone Morphogenetic Protein Receptor type II (*BMPR2*) and insulin receptor substrate 1 (*IRS1*) were correlated to STO and FTO muscle fibers. MiR-25 has been documented to be abundant in cardiomyocytes. It targets the mitochondrial calcium uniporter (MCU) and Ca^2+^ transporting ATPase (ATP2A2) and plays a role in cardiac contractility [75, 76]. In the present study, miR-25 was proposed to target both genes *BMPR2* and *IRS1. BMPR2* encodes a member of the bone morphogenetic protein receptor family of transmembrane serine/threonine kinases. It is essential for BMP signaling and may be involved in the regulation of adipogenesis and hence in obesity [51]. IRS1 is a major molecule mediating insulin-signaling pathways. Insulin not only regulates stimulation of protein synthesis and glucose storage [77], but also has effect on mitochondrial function and oxidative capacity of skeletal muscle via increasing the expression level of complex I and complex IV and hence ATP production [78]. MiR-363 and its target gene ubiquitin specific peptidase 24 (*USP24*) were correlated to STO fibers, mitochondrial respiratory activity including state 3 pyruvate and state 3 succinate, and AMP concentration in muscle cells, while miR-28 and its target gene *HECW2* were correlated to STO muscle fibers. USP24 belongs to a large family of cysteine proteases that function as deubiquitinating enzymes. *HECW2* encodes HECT, C2 and WW domain containing E3 ubiquitin protein ligase 2 which is a major component of ubiquitin proteasome system (UPS). UPS utilizes ATP to promote protein degradation and regulate muscle mass. Accumulated ubiquitin proteins in fast- to slow- transforming rabbit muscle revealed a possible role of UPS in muscle fiber specificity [79]. Interestingly, miR-363 has been discovered as a negative regulator of adipogenesis as described previously [46]. Misregulation of miRNAs belonging to miR-23a/27a/24-2 cluster has been recently associated to hypertrophic cardiomyopathy and skeletal muscle atrophy [80]. MiR-27 was almost sixfold greater in slow-twitch than in fast-twitch muscle in vivo. It posttranscriptionally regulates fast-specific myostatin (MSTN) expression, which mature mRNA level is sixfold greater in fast vs slow muscle [81]. In this study, miR-27 was identified to be associated with PFK activity – a rate-limiting enzyme in glycolysis and potentially target cytochrome P450, family 24, subfamily A, polypeptide 1 (*CYP24A1*), which catalyzes the side-chain oxidation of vitamin D [82]. The vitamin D pathway has the suppressive effect on brown adipocyte differentiation and mitochondrial respiration [83]. MiR-210 and its predicted targets *ATP5I*, *ME3*, *MTCH1* and *CPT2* were highly correlated to ADP and ATP concentration in present study. MiR-210 modulates mitochondrial function, decreases COX10 expression and activates the generation of reactive oxygen species (ROS) [14]. *ATP5I* encodes ATP synthase mitochondrial F0 complex subunit E and it is required for the full assembly of the ATP synthase and ATP production [84]. *ME3* encodes mitochondrial NADP (+)-dependent malic enzyme 3. The regulation of human mitochondrial NADP (+)-dependent malic enzyme by ATP and fumarate may be crucial for the metabolism of glutamine for energy production [85]. *MTCH1* and *CPT2* encode for mitochondrial carrier 1 and carnitine palmitoyltransferase 2 respectively. MTCH1 also known as Presenilin 1-associated protein (PSAP) which acts as a proapoptotic mitochondrial protein induce apoptosis independent of the proapoptotic proteins Bax and Bak [86]. The two isoforms of MTCH1 share two proapoptotic domains and multiple internal signals for import into the mitochondrial outer membrane [87]. Fatty acid is a major energy source for the heat and skeletal muscle. CPT2 together with CPT1 are involved in beta-oxidation of long chain fatty acids in the mitochondria [88]. Altogether suggests that miR-210 and its target genes *ATP5I*, *ME3*, *MTCH1* and *CPT2* are likely to be involved in ATP production, apoptosis and beta-oxidation of long fatty acids in mitochondria. We have demonstrated that correlation relationship between miRNA and target mRNA can be used to functionally link to phenotypes of interest such as muscle fiber type specification, mitochondrial respiration activity and metabolic enzymes related to ATP production.

### Crosstalk between mitochondria and UPS in skeletal muscle

Up to now we have shown that the identified miRNA-mRNA networks are linked to muscle fiber types, oxidative enzyme activities and ATP generation. Some of these target genes are involved in mitochondrial function and UPS. An interesting finding was the significant correlation between mitochondrial and UPS related gene expressions. More specifically, mitochondrial related genes including *ATP5I*, *ME3*, *MTCH1*, *CYP24A KIAA1279*, *PHB2*, *PDHA1* and *UQCC* were highly correlated to at least one of the UPS-related genes including *HECW2*, *USP24*, *UBFD1*, *MUL1*, *APP* and *HSPA2*.

The tightly interdependent relationship between mitochondria and UPS system has been described in many age-related diseases such as Alzheimer’s and Parkinson’s disease [89–91]. Our present study revealed a link between these two systems at the level of gene expression under normal state, since all the investigated animals were healthy. In other words, both mitochondria and UPS might contribute to energy metabolism of skeletal muscle via fine-turning the gene expression by miRNAs under physiological conditions.

The HECT, C2 and WW domain containing E3 ubiquitin protein ligase 2 (HECW2), ubiquitin specific peptidase 24 (USP24), ubiquitin family domain containing 1 (UBFD1) and mitochondrial ubiquitin ligase activator of NFKB 1-like (MUL1), Amyloid beta precursor protein (APP) and Heat shock 70 kDa protein 2 (HSPA2) are either the major components or associated with the UPS system [92, 93]. Those genes targeted by several miRNAs including miR-28, miR-363, miR-2020, miR-24, miR-1207, miR-345 and miR-58 may be the cause of fluctuation of the UPS degradation for ubiquitin proteasome-dependent molecules [94] such as transcriptional coactivator PGC-1α which acts as a master regulator for mitochondrial biogenesis, to control mitochondrial gene expression indirectly. On the other side, miR-210 and miR-885 targeted genes of ATP synthase mitochondrial F0 complex subunit E (ATP5I), Pyruvate dehydrogenase alpha 1 (PDHA1) and Ubiquinol-cytochrome c reductase complex chaperone (UQCC) [95] could affect cellular ATP generation, followed by influencing the ATP-dependent UPS system [96]. However, further detail information of the interaction between mitochondria and UPS still remains elusive.

## Conclusion

In this study, we modelled the miRNA-mRNA regulatory networks related to muscle fiber type, metabolic enzyme activity and ATP production using the correlation information between expressed miRNAs and target mRNAs as well as muscular phenotypic measurements of Duroc and PiNN pigs. These complex networks may contribute to the muscle phenotypic variations by fine-tuning the expression of genes. Altogether, the results provide an insight into a regulatory role of miRNAs in muscular energy metabolisms.

## Abbreviations

miRNA, microRNA; STO, slow-twitch-oxidative; FTO, fast-twitch-oxidative; FTG, fast-twitch-glycolytic; PiNN: pietrain; UPS, ubiquitin proteasome system.

## Additional files

Additional file 1: Definition and measurement of phenotypical traits. (XLSX 11 kb) Additional file 2: The primer sequences in qRT-PCR. (XLSX 10 kb) Additional file 3: Differentially expressed miRNAs in porcine LD muscle between Duroc and Pietrain breed types at adult stage. (XLSX 17 kb) Additional file 4: Different expressed miRNA-mRNA pairs between Duroc and PiNN after target prediction. (XLSX 130 kb) Additional file 5: Individual miRNAs and their correlated phenotypes for Duroc and PiNN. (XLSX 20 kb) Additional file 6: Individual mRNAs and their correlated phenotypes for Duroc and PiNN. (XLSX 18 kb) Additional file 7: Phenotype correlated miRNA-mRNA pairs after target prediction for Duroc and PiNN. (XLSX 48 kb)
